# Minimal and Direct Access Surgery in Urology

**DOI:** 10.1155/DTE.3.99

**Published:** 1996

**Authors:** D. L. Repassy, D. Frang, G. J. Jako

**Affiliations:** 1 Department of Urology St. Stephen Hospital Budapest Hungary; 2 Department of Urology Semmelweis University of Medicine Budapest Hungary; 3 Department of Otolaryngology Boston University School of Medicine USA; 4 H-1096 Nagyvarad ter 1. Budapest Hungary; 5 169 East Emerson Street Melrose-Boston MA 02176 USA

## Abstract

An alternative method to laparoscopic surgery has been developed for urological procedures. The surgery is minimal access because the length of the single skin incision ranges from 3–6 cm depending on the type of operation. It is direct access because the surgeon sees the operative area directly and stereoscopically by eye without video-optical support. The procedure requires a special open-lumen retractorscope (Jakoscope^TM^) with a high intensity fiberoptic light system and modified standard hand instruments. Among the procedures performed nephrectomy, ureterolithotomy, prostatic adenomectomy, spermatic vein ligation and others have been performed. The kidney procedures have been operated retroperitoneally through a minilumbotomy incision. The procedures are simple, rapid and the instruments are inexpensive. The postoperative pain and morbidity are comparable to the laparoscopic approach.


*Diagnostic and Therapeutic Endoscopy*, 1996, Vol. 3, pp. 99–105
Reprints available directly from the publisher
Photocopying permitted by license only

© 1996 OPA (Overseas Publishers Association)
Amsterdam B.V. Published in The Netherlands
by Harwood Academic Publishers
Printed in Malaysia

### Minimal and Direct Access Surgery in Urology

D. L. REPASSY[a,*], D. FRANG[b], and G. J. JAKO[c]

[a]*Department of Urology, St. Stephen Hospital, Budapest, Hungary,* [b]*Department of Urology, Semmelweis University of Medicine, Budapest, Hungary,* [c]*Department of Otolaryngology, Boston University School of Medicine*

*(Received 3 July 1995; Revised 27 May 1996; In final form 24 June 1996)*

**An alternative method to laparoscopic surgery has been developed for urological procedures. The surgery is minimal access because the length of the single skin incision ranges from 3–6 cm depending on the type of operation. It is direct access because the surgeon sees the operative area directly and stereoscopically by eye without video-optical support. The procedure requires a special open-lumen retractorscope (Jakoscope™) with a high intensity fiberoptic light system and modified standard hand instruments. Among the procedures performed nephrectomy, ureterolithotomy, prostatic adenomectomy, spermatic vein ligation and others have been performed. The kidney procedures have been operated retroperitoneally through a minilumbotomy incision. The procedures are simple, rapid and the instruments are inexpensive. The postoperative pain and morbidity are comparable to the laparoscopic approach.**

*Keywords:* Surgery—urogenital, minimally invasive surgery

### INTRODUCTION

In recent years, minimally invasive videolaparoscopic procedures have been applied more and more to urological procedures [1,2,3]. In these multiple skin incisions in the range of 5–30 mm or more are utilized. If the operation is performed through the peritoneum, $CO_2$ insufflation is used with its potential complications.

The widening list of indications for laparoscopic surgery, surgical techniques, and results have been widely covered in urological publications [4,5,6,7,8,9].

The transurethral, endourological, and percutaneous methods are a form of minimal trauma procedures. Organ removal by laparoscopy such as nephrectomy and partial nephrectomy compared to classical open procedures requires prolonged operating and anesthesia time. In addition, the expense of videolaparoscopic equipment and disposable endoscopic instrumentation make this procedure expensive. Surgeons are required to learn a completely different technique using 2-dimensional video monitors with a prolonged learning curve. During the initial learning, the frequency of complications are higher compared to the classical wide open operation.

We looked for a new viable alternative. From 1992, first experimentally in porcines and in fresh human

*Corresponding author.
Present address: H-1096 Nagyvarad ter 1., Budapest, Hungary or G. J. Jako, M.D., 169 East Emerson Street, Melrose-Boston, MA 02176.

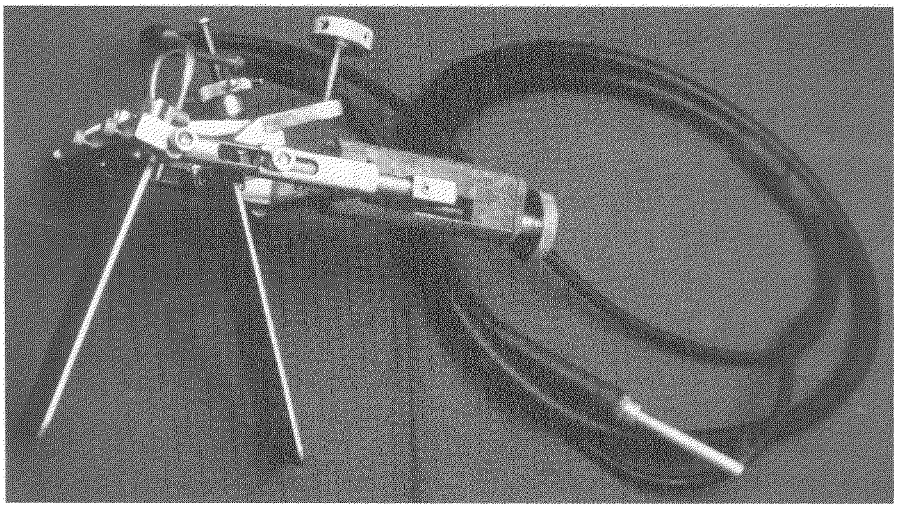

FIGURE 1 Retractorscope Jakoscope™ I with fiberoptics illumination.

cadavers, then in clinical cases, we started to develop new and different minimally invasive procedures. Here, we report on our favorable experiences.

### MATERIALS AND METHODS

### Instruments

For the surgery, a retractorscope, (Jakoscope™) [10]* is used. It is an open lumen large endoscope. It has 2 blades which can be rotated and the distance between the blades can be changed with a rack and pinion device. The blades are interchangeable and available with different widths form 20 to 60 mm and lengths from 6 to 16 cm. A bifurcated large fiberoptic cable is used for illumination which is an integral part of the instrument. (Fig. 1) The high intensitiy lighting is provided by a 300W metal halide or xenon light source. Through this open lumen endoscope, the surgeon has stereoscopic view and direct access to the operating field. Since the blades can be rotated outward, the surgical field can be made larger than the incisional window through the skin. The instruments for manipulation are modifications of conventional ones but angled to keep the surgeons hands from obstructing the view (Fig. 2). For the surgery, no expensive laparoscopic or disposable instruments are needed. An optional color microvideo camera can be used for demonstration or video recording.

*Atlantis Surgical, New Brunswick, NJ 08901.

### Patients

#### *Nephrectomy*

Nine patients have undergone minimal and direct access nephrectomies in our department. Three had nonfunctioning hydronephrotic kidneys, 2 had renal hypoplasia and 4 had chronic pyelonephritis and secondary hypertension. The kidney removal is done according to the following technique: the patient is placed in lateral position. General anesthesia is administered. A single 5-cm long oblique incision is made near the 12th rib as a minilumbotomy. The muscle layers are dissected with minimal trauma and the retroperitoneal space is exposed. The retractorscope is then inserted and by opening the blades slowly, it creates a retroperitoneal space. The Gerota's fascia is transected. The renal vessels and the ureter are identified, ligated, and cut similarly as in conventional classic nephrectomies. During the preparation, a large angled Allis-type clamp is used to hold the kidney. It

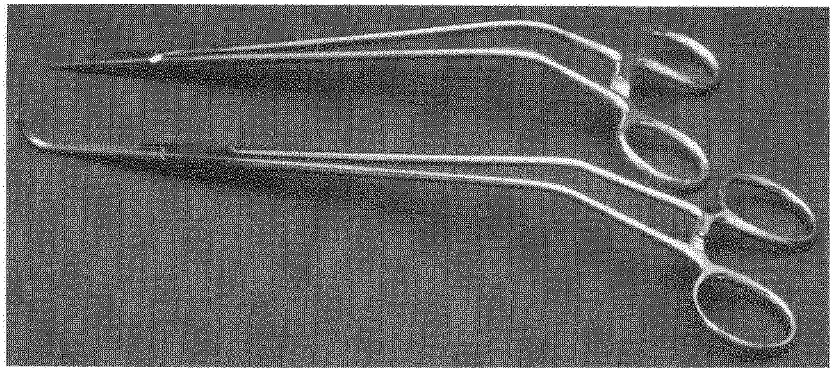

FIGURE 2 Modified-curved-hand instruments.

is removed intact through the retractorscope without morcellation. Drainage and wound closure is done in a conventional manner.

### *Ureterolithotomy*

Six patients were operated on for ureteric stones because of failed treatment. The minimally invasive ureterolithotomy was done according to the following method, the patient is positioned on the operating table; a 3-cm long flank skin incision is made under general anesthesia. The muscles are dissected with minimal trauma and the retractorscope is inserted in the retroperitoneal space. Moving and rotating the retractor blades, the ureter is exposed. It is then carefully lifted, palpated, and incised. The stone is removed and the incision and the soft tissues are closed as in conventional surgery.

### *Spermatic Vein Ligature*

Five patients underwent retroperitoneal ligation of varicocele because of symptomatic varicocele and infertility. The operation was done in a conventional fashion but through a short incision with the retractorscope. A 3-cm long skin incision is made medial to the anterior superior iliac spine to expose the retroperitoneal space. After inserting the retractorscope, the spermatic cord is identified. The internal spermatic artery and ductus deferens are isolated and spared. The spermatic vein is gently dissected and ligated (or clipped with endoclips) and divided. Closing the wound is conventional.

### *Prostatic Adenomectomy—Transvesical*

Twenty-seven patients with benign prostatic hyperplasia (BPH) were adenomectomized according to the transvesical method using the retractorscope. A transverse, suprapubic 5-cm long skin incision is made and the bladder is exposed. The bladder is incised and the retractorscope is inserted into it. The adenomectomy enucleation is performed conventionally as in the classical method.

### RESULTS

### Patients

### *Nephrectomy*

Nine patients (3 men and 6 women) have undergone retractorscopic nephrectomy in our department. The patient's data and indications are listed in Table I. The average operating time was 85 minutes (range 55 to 125). Average estimated blood loss was 115 cc (range 0 to 300 cc). On average, patients required 14 mg of morphine sulfate (range 0 to 40) for postoperative pain; 5 patients did not request any pain medication. The average hospital stay was 6 days (range 5 to 7). There were no intraoperative or postoperative complications.

Fifteen patients who underwent conventional, classical nephrectomy were compared, as a control group, to the minilumbotomy nephrectomy patients. Indications for nephrectomy were the same in both

TABLE I Minilaparotomy nephrectomy

| Pt. | Sex | Age | Indication | Operating time (min.) | Blood Loss (cc.) | Postop-analg. (morph. mg.) | Hosp. stay days |
|---|---|---|---|---|---|---|---|
| NK | F | 38 | Hydronephrosis | 105 | 300 | 30 | 7 |
| KM | M | 55 | Chronic pyelonephritis | 125 | 250 | 40 | 7 |
| BG | F | 27 | Hypoplasia | 55 | — | — | 5 |
| SI | F | 48 | Hypertonic kidney | 95 | 65 | — | 5 |
| NVJ | M | 32 | Hypertonic kidney | 80 | 100 | 20 | 5 |
| NI | M | 47 | Hypertonic kidney | 65 | — | — | 5 |
| MK | F | 52 | Hydronephrosis | 115 | 180 | 40 | 7 |
| CSL | F | 35 | Hydronephrosis | 65 | 140 | — | 7 |
| SL | F | 28 | Hypoplasia | 60 | — | — | 6 |
| Avg. | | 40 | | 85 | 115 | 14 | 6 |

groups. Tumorous and traumatic cases were excluded from this series. The data of the control group for comparison are shown in Table II.

The operating time was initially slightly longer in the minilumbotomy group. There was no significant difference between blood loss in the two groups. The hospital stay was shorter in the retractorscopic nephrectomy patients, and they required less postoperative pain medication.

### *Ureterolithotomy*

Six patients have undergone minimally invasive ureterolithotomy applying microlumbotomy incision (Table III). The average operating time was 44 minutes (range 35 to 65 minutes). There was no blood loss in the retractorscopic ureterolithotomy patients except one case—100 cc. Only one patient required an analgesic after surgery. The average hospital stay was 5 days.

In the control group, there were 16 patients who were operated on earlier by the conventional open method. The data for comparison are in Table IV.

The operating time and the blood loss were less, but the hospital stay of the conventional ureterolithotomy patients was longer. In addition, the retractorscopic ureterolithotomy patients did not require postoperative pain medication.

### *Spermatic Vein Ligature*

Our 5 cases are not appropriate for analysis of the effectiveness of this method. The retroperitoneal ligature of the testicular vein was easily accomplished through the retractorscope. There were no intraoperative complications.

### *Prostatic Adenomectomy*

Table V summarizes the data of prostatectomy patients and the control group of classical transvesical adenomectomy. The data does not show significant differences except in the retractorscopic cases where the skin incision in 5 cm patients had less incisional pain compared to the 15 cm conventional incision.

TABLE II Comparison of classical open versus retractorscopic nephrectomy

| | Classical nephrectomy | Retractorscopic nephrectomy |
|---|---|---|
| Number of patients | 15 | 9 |
| Average age (range) | 44 (26 to 61) | 40 (24 to 56) |
| Average operating time (minutes) | 55 (35 to 100) | 85 (55 to 125) |
| Postoperative analgesia (morphine mg.) | 87 (60 to 140) | 14 ( 0 to 40) |
| Average blood loss (cc.) | 105 ( 0 to 300) | 115 ( 0 to 300) |
| Average days hospital stay (range) | 11 ( 7 to 14) | 6 ( 5 to 7) |

TABLE III Minilaparotomy Ureterolithotomy

| Pt. | Sex | Age | Indication | Operating time (min.) | Blood loss (cc.) | Postop-analg. (morph. mg.) | Hosp. stay days |
|---|---|---|---|---|---|---|---|
| LP | M | 56 | Impacted stone | 35 | — | — | 5 |
| KE | M | 62 | Impacted stone | 45 | — | — | 5 |
| BL | M | 59 | Impacted stone | 40 | — | — | 5 |
| TJ | M | 67 | Impacted stone | 65 | 100 | 40 | 7 |
| KA | F | 59 | Impacted stone | 40 | — | — | 5 |
| NI | F | 48 | Impacted stone | 35 | — | — | 5 |
| Avg. | | 40 | | 44 | — | — | 5 |

### DISCUSSION

In regard to the short single incisions used in these operations, Rozsos and Jako classified these for cholecystectomies up to 4 cm as microlaparotomy, up to 6 cm as modern minilaparotomy and up to 10 cm as classical minilaparotomy [11]. Similarly, we may use such terms as microlumbotomy and minilumbotomy.

Bozzini presented his open-lumen endoscope in 1806 in Germany. In 1879, Nietze built the first optical telescopic instrument for cystoscopy. In the following years, open-lumen endoscopes had been developed for laryngoscopy, bronchoscopy, esophagoscopy and rectosigmoidoscopy [12].

Open surgery requires a long incision because the standard operating room light cannot adequately illuminate the surgical field through a small operating window. Also, the surgeon's head and hands would block the light and the view with a small opening. Because of the wide exposure in classical surgery, the surgeon keeps his hands free and manipulates the instruments more from inside the body than from the outside. Our techniques and instruments eliminate these problems.

For our work, we use a large open-lumen type endoscope with high intensity fiberoptic lighting and bivalve retractor blades. To avoid confusion, it was named "retractorscope" or alternatively after its inventor, Jakoscope™. Besides urology it is in clinical use in 6 other surgical specialties.

Urology was an early surgical specialty for minimally invasive or minimal trauma surgery. Cystoscopy eliminated the major trauma of opening the bladder for certain diseases. It was then followed by percutaneous nephrostomy using a minimal access opening. Further technical advances and instruments made endourology a reality. These contributed in making the procedures minimally invasive compared to open operations.

When laparoscopic surgery gained momentum in various surgical specialties, it started to be referred to as a minimally invasive, minimal trauma, or minimal access procedure. Presently, all of these terms denote laparoscopic surgery.

In laparoscopic surgery, $CO_2$ insufflation is used to gain space in the abdomen by "gas retraction", i.e., to move the intestines away. It is not without potential complications or even fatality especially in high risk

TABLE IV Comparison of data for conventional open versus minilaparotomy ureterolithotomy

| | Conventional ureterolithotomy | Retractorscopic ureterolithotomy |
|---|---|---|
| Number of patients | 16 | 6 |
| Average age (range) | 52 (31 to 65) | 47 (26 to 61) |
| Average operating time (minutes) | 50 (30 to 70) | 44 (35 to 65) |
| Postoperative analgesia (morphine mg.) | 76 (0 to 135) | — |
| Average blood loss (cc.) | — | — |
| Average days hospital stay (range) | 9 (8 to 10) | 5 (4 to 7) |

TABLE V Minilaparotomy prostatic adenomectomy

| | Retractorscopic prostatic adenomectomy | Conventional prostatic adenomectomy |
|---|---|---|
| Number of patients | 27 | 52 |
| Average age (range) | 67 (55–81) | 65 (54–82) |
| Average operating time (minutes) | 56 (40–70) | 54 (40–70) |
| Average analgesia (morphine mg.) | 96 (0–152) | 104 (0–164) |
| Average blood loss (cc.) | 880 (600–1500) | 940 (600–1600) |
| Average days hospital stay (range) | 8 (7–10) | 9 (7–14) |

elderly patients [13] The use of multiple small openings with trocars may reduce trauma compared to standard incision but the total length of the incisions for complicated procedure may add up. For example, one 5 mm, three 10 mm, and one 15 mm trocar incisions add up to 50 mm. In case of a nephrectomy for disease or for transplantation another 50 to 100 mm incision may be added [14,15].

Some urologic laparoscopic techniques use transperitoneal entry which may add the risk of peritoneal complication. Numerous vascular accidents have been reported involving larger arteries or veins [16,17]. These cannot be handled laparoscopically. In these cases, a rapid decision and conversion into classical open surgery is required.

The learning curve of laparoscopic surgery is long, since the surgeon has to re-educate himself to work from a 2-dimensional video monitor. He manipulates new types of instruments which require different motions and coordination than he has been used to. He is deprived of the 3-dimensional stereoscopic vision to view the surgical area by eye.

The number of patients for urologic laparoscopic operations is much smaller compared to gallbladder surgery. Therefore, the time for the surgeon to gain adequate experience is longer [18]. The surgical anatomy is more complicated because of large blood vessels which prolong the learning curve. These factors increase the operating and anesthesia time, which make these procedures very expensive especially when many disposables are added [19]. The prolonged operation, anesthesia, and $CO_2$ insufflation is also less gentle on elderly patients. These are some of the reasons why after 5 years, urologic laparoscopic procedures are practiced at fewer hospital than cholecystectomies. Some authors still consider them experimental [20].

Here we are offering a new surgical concept, Minimal and Direct Access (MDA), instrumentation and techniques as an alternative to some classical and most laparoscopic procedures. MDA™ surgery offers the following advantages compared to classical open surgery:

1. Minimal surgical trauma
2. Less postoperative pain
3. Shorter hospital stay

Compared to laparoscopic surgery:

1. Single incision
2. Shorter operating and anesthesia time
3. No intraperitoneal invasion or gas insufflation
4. Inexpensive reusable instrumentation
5. Direct stereoscopic 3-dimensional visualization
6. No optical video camera required for surgery (except for demonstration and recording)
7. Postoperative pain is comparable to laparoscopy
8. Direct palpation, easy ligation and suturing
9. Easy application of operating microscope and lasers
10. The potential to expand this technique to other urological procedures including the treatment of benign and certain malignant tumors.

### CONCLUSION

A new surgical concept, instruments and techniques are described for urological surgery. It offers minimally invasive alternatives to certain types of open urological operations and several benefits over laparoscopic procedures.

We recommend our MDA approach, in which the surgeon sees everything the same way as he is used to in classical operations stereoscopically with his own two eyes. He is also able to palpate, suture and ligate directly through the single minilumbotomy or minilaparotomy incision.

### *References*

[1] Wong, H. Y. and Griffith, D. P. (1994). Laparoscopy; Editorial; *J. Urol.*, **151**, 938.

[2] Clayman, R. V., Kavoussi, L. R., Soper, N. J., Dierks, S. M., Meretyk, S,. Darcy, M. D., Roemer, F. D., Pingleton, E. D., Thomson, P. G. and Long, S. R. (1991). Laparoscopic nephrectomy: initial case report. *J. Urol.*, **146**, 278.

[3] Clayman, R. V., Kavoussi, L. R., Soper, N. J., Albals, D. M., Figenshau, R. S. and Chandhoke, P. S. (1992). Laparoscopic nephrectomy: review of the initial ten cases. *J. Endourol.*, **6**, 127.

[4] Gaur, D. D., Agarwal, D. K. and Purohit, K. C. (1993). Retroperitoneal laparoscopic nephrectomy: initial cases report. *J. Urol.*, **149**, 103.

[5] Gaur, D. D. (1992). Laparoscopic operative retroperitoneoscopy: use of a new device. *J. Urol.*, **148**, 1137.

[6] Gaur, D. D. Agarwal, D. K., Purohit, K. C., Darshane, A. S. (1994). Retroperitoneal laparoscopic pyelolithotomy. *J. Urol.*, **151**, 927.

[7] Raboy, A., Ferzli, G. S., Ioffreda, R. and Albert, P. S. (1992). Laparoscopic ureterolithotomy. *Urology*, **39**, 223.

[8] Gangal, H. T., Gangal, P. H. and Gangal, M. H. (1993). An attempt at a percutaneous retroperitoneoscopic approach to ureterolithotomy. *Surg. Endosc.*, **7**, 455–458.

[9] Bellman, G. C. and Smith, A. D. (1994). Special considerations in the technique of laparoscopic ureterolithotomy. *J. Urol.*, **151**, 146–149.

[10] Jako, G. J., Retractor and method for direct access endoscopic surgery, U.S. Patent #5, 503 617, April 2; 1996.

[11] Rozsos, I., Jako, G. J. (1995). Microlaparotomy cholecystectomy (letter), *Ann. Surg.*, **222**, 762–763.

[12] *Encyclopedia of Medicine*, American Medical Association, 1989.

[13] Folger, W. H., Clayman, R. V., McDougall, E. M. *et al.* (1996). Carbon dioxide and helium insufflation during laparoscopy, *J. Urol.*, **155 suppl.**, 656A.

[14] Suzuki, K., Ishikawa, A., Kageyama, S. *et al.* (1996). Transperitoneal laparoscopy—assisted live donor nephrectomy, *J. Urol.*, **155 suppl.**, 298A.

[15] Suzuki, K. Masuda H., Ushiyama, T. *et al.* (1995). Gasless laparoscopy assisted nephrectomy without tissue morcellation for renal carcinoma, *J. Urol.*, **154**, 1685–7.

[16] Thiel, R., Adams, J. B., Schulam, P. G., Moore, R. G., Kavoussi, L. R. (1996). Venous Dissection injuries during laparoscopic urologic surgery, *J. Urol.*, **155 suppl.**, 659A.

[17] Gill, I. S., Kavoussi, L. R., Clayman, R. V. *et al.* (1994). Complications of laparoscopic nephrectomy, *J. Urol.*, **151 suppl.**, 461A.

[18] See W. A., Cooper, C. S. Fisher, R. J. (1993). Predictors of laparoscopic complications after formal training in laparoscopic surgery, *JAMA*, **270**, 2689–92.

[19] Winfield, H. N., Rashid, T. M., Lund, G. O. *et al.* (1994). Comparative financial analysis of laparoscopic versus open nephrectomy, *J. Urol.*, **151 supp.**, 342A.

[20] Weber, H. M. Schneider, A. C., Drautscheck, A. W. *et al.* (1996). Hazards of laparoscopic urology, *J. Urol.*, **155 suppl.**, 300A.

